# Nanomolar EP4 receptor potency and expression of eicosanoid-related enzymes in normal appearing colonic mucosa from patients with colorectal neoplasia

**DOI:** 10.1186/s12876-022-02311-z

**Published:** 2022-05-12

**Authors:** Ulrike Ries Feddersen, Sebastian Kjærgaard Hendel, Mark Alexander Berner-Hansen, Thomas Andrew Jepps, Mark Berner-Hansen, Niels Bindslev

**Affiliations:** 1 Digestive Disease Center, Bispebjerg Hospital, 2400 Copenhagen NV, Denmark; 2 Department of Biomedical Sciences, University of Copenhagen, 2200 Copenhagen N, Denmark

**Keywords:** Colorectal cancer, EP receptors, mRNA expression, Short circuit current, Lipoxygenase

## Abstract

**Background:**

Aberrations in cyclooxygenase and lipoxygenase (LOX) pathways in non-neoplastic, normal appearing mucosa from patients with colorectal neoplasia (CRN), could hypothetically qualify as predisposing CRN-markers.

**Methods:**

To test this hypothesis, biopsies were obtained during colonoscopy from macroscopically normal colonic mucosa from patients with and without CRN. Prostaglandin E2 (PGE_2_) receptors, EP1-4, were examined in Ussing-chambers by exposing biopsies to selective EP receptor agonists, antagonists and PGE_2_. Furthermore, mRNA expression of EP receptors, prostanoid synthases and LOX enzymes were evaluated with qPCR.

**Results:**

Data suggest that PGE_2_ binds to both high and low affinity EP receptors. In particular, PGE_2_ demonstrated EP4 receptor potency in the low nanomolar range. Similar results were detected using EP2 and EP4 agonists. In CRN patients, mRNA-levels were higher for EP1 and EP2 receptors and for enzymes prostaglandin-I synthase, 5-LOX, 12-LOX and 15-LOX.

**Conclusions:**

In conclusion, normal appearing colonic mucosa from CRN patients demonstrates deviating expression in eicosanoid pathways, which might indicate a likely predisposition for early CRN development and furthermore that PGE_2_ potently activates high affinity EP4 receptor subtypes, supporting relevance of testing EP4 antagonists in colorectal neoplasia management.

**Supplementary Information:**

The online version contains supplementary material available at 10.1186/s12876-022-02311-z.

## Background

Colorectal cancer (CRC) is the third most common type of cancer worldwide and the second leading cause of cancer related deaths [1]. Adenocarcinomas constitute the majority of CRC and the carcinogenesis of this type of CRC is a multifactorial process, in which an accumulation of mutations leads to the formation of colorectal neoplasia (CRN), initially as benign adenomas and subsequently malignant adenocarcinomas [2]. Genetics and chronic colonic inflammation are known risk factors for developing CRC [3], involving altered activity of the arachidonic acid (AA) metabolism including prostaglandins. The specific mechanisms, however, are poorly understood.

**Fig. 1:**
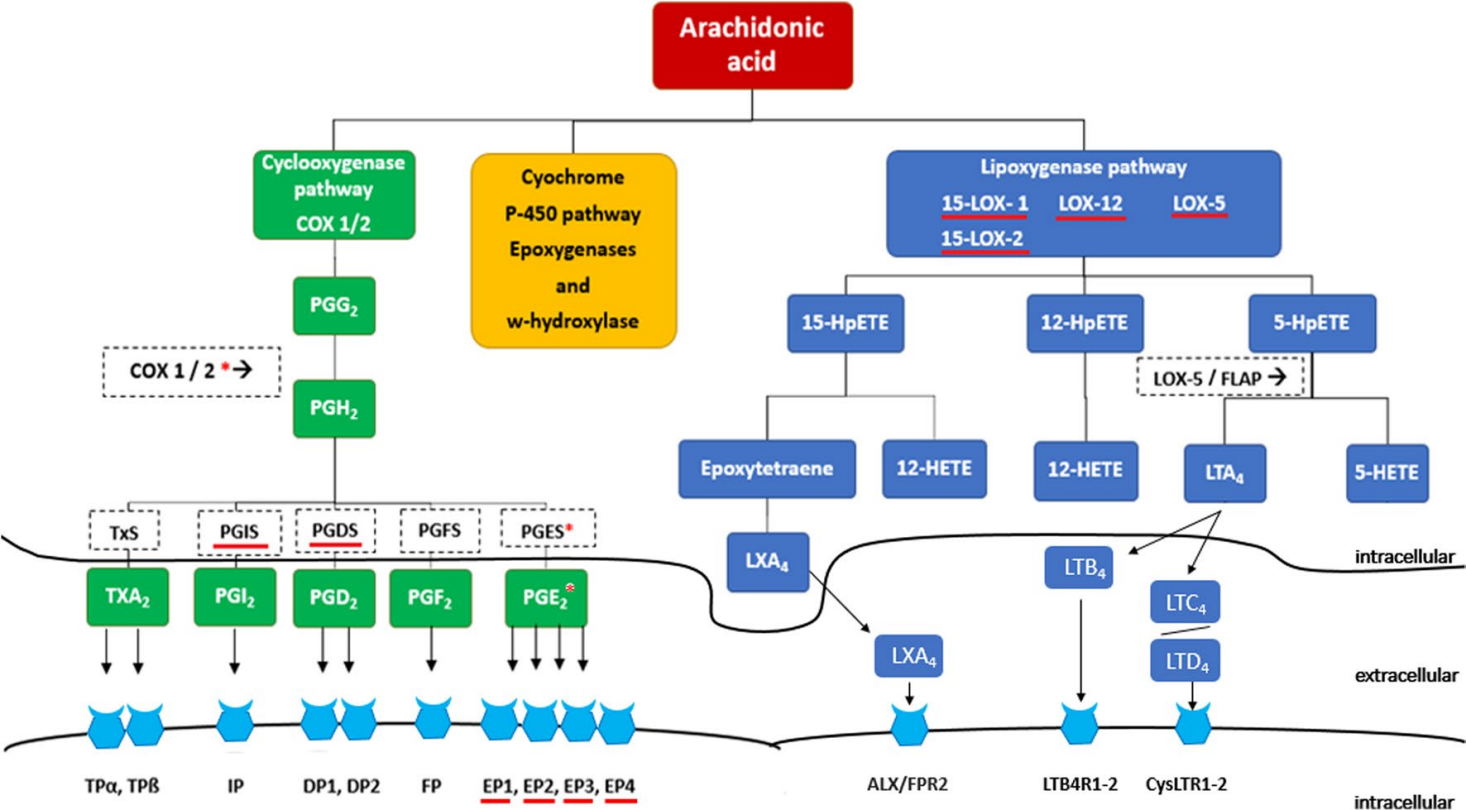
Model of the metabolization of arachidonic acid (AA). AA is metabolized by 3 different groups of enzymes: cyclooxygenases (COX), lipoxygenases (LOX) and epoxygenases (cytochrome P450). The COX pathway consists of 2 isozymes: COX-1 and COX-2. Both isozymes metabolize AA into PGG_2_ and then into PGH_2_, which is further converted to the prostaglandins (PGs) PGD_2_, PGE_2_, PGF_2α_, PGI_2_ and thromboxane A_2_, (TXA_2_) by their respective synthases [3]. Each product binds to its specific membrane receptor. The CYP-450 pathway converts AA by epoxygenases and ω-hydroxylase into other downstream products, not shown. The LOX pathway consists of 3 main enzymes termed 5-LOX, 12-LOX and 15-LOX (isozymes 15-LOX-1 and 15-LOX-2). They metabolize AA into hydroperoxyl-eicosatetraenoic acids (HPETEs), which are further reduced to hydroxyeicosatetraenoic acids (HETEs). The 5-LOX enzyme differs by also metabolizing 5-HPETE into leukotriene A_4_ by means of 5-lipoxygenase-activating protein (FLAP). *Enzymes already investigated in our laboratory; data published. Receptors/enzymes investigated in this study are underlined with red

Non-steroid anti-inflammatory drugs (NSAIDs), as aspirin (acetylsalicylic acid), and non-selective cyclooxygenase (COX) inhibitors ameliorate CRC development [4, 5]. NSAIDs attenuate the inflammatory response mainly by inhibiting enzyme activity of COX isozymes, COX-1 and COX-2, thus preventing conversion of AA into the prostanoids PGD_2_, PGE_2_, PGF_2α_, PGI_2_ and thromboxane A_2_ (TXA_2_), Fig. 1, [3].

COX-2 expression is elevated in human adenomas as well as in adenocarcinomas, which is why COX-2 is believed to be central to CRN and CRC pathogenesis [6]. Accordingly, the protective effect of NSAIDs on CRC development is likely due to a reduced COX-activity as well as associated PGE_2_ production [3, 5, 7].

PGE_2_ elicits tumorigenic effects by binding to either of its 4 G-protein coupled surface receptors, EP1-4, Fig. 1 [8]. These effects include proliferation, migration, invasion and angiogenesis [8]. Each of the receptor subtypes has been linked to CRC tumorigenesis using knock-out mice [9–11]. In particular, EP4 is suspected to be of special tumorigenic importance due to its activation of several central kinases [12, 13].

For the remaining prostanoids; TXA_2_ is considered mainly tumorigenic, PGI_2_ anti-tumorigenic and PGF_2_ and PGD_2_ have uncertain tumorigenic roles [14, 15].

Recently, another AA-related pathway, the lipoxygenase (LOX) pathway, was suggested to be associated with CRC. Particularly the enzymes 5-LOX, 12-LOX and 15-LOX and its isoforms (15-LOX-1 and 15-LOX-2) appear to be involved [16, 17]. Unlike the COX pathway, the end products of LOX enzymes are hydroxyeicosatetraenoic acids (HETEs) derivates, Fig. 1. Current evidence suggests a pro-tumorigenic effect of 5-LOX and 12-LOX metabolites in CRC, whereas 15-LOX-1 and 15-LOX-2 are mainly classified as anti-tumorigenic and downregulated in CRC tissue [16, 17].

Several theories in form of “field effects” and “mutator pathways” for primary tumor-induced changes in near and distant gene expression have been forwarded over the last 70 years [18–20]. It remains unsolved whether tumor-adjacent imbalances in eicosanoid-related enzymes and/or receptors are inherited initiating factors, a predisposition, rather than consequences of a nearby tumor’s neoplastic “field effect”.

Here we hypothesize that genetically inherited constructs in eicosanoid signaling might be an individual early CRC tumorigenic predisposition detectable in macroscopically normal appearing tissue. Accordingly, we examined eicosanoid-related enzymes and receptors in non-neoplastic colonic mucosa both from patients with and without CRN. Specifically, we characterized function and expression of the EP receptor subtypes and examined the expression levels of prostaglandin D2 synthase (PTGDS), prostaglandin I2 synthase (PTGIS) and the PGF_2_α- reductase AKR1B1 (an aldo–keto reductase), all as indicators for altered levels of their respective prostanoids [21]. Finally, we determined expression levels of 5-, 12-, and 15-LOX enzymes. Both the actual and former eicosanoid-related entities, studied for function and expression by us, are labeled in Fig. 1.

## Methods

### Study population

White Danish patients (45–80 years of age) referred for colonoscopy on suspicion of colorectal disease (e.g. positive fecal occult blood test or persistent abdominal discomfort), were screened for participation. Exclusion criteria included history of inflammatory bowel disease, conditions of intestinal malabsorption (e.g. coeliac disease and lactose intolerance), familiar risk of CRC (hereditary nonpolyposis colorectal cancer and familial adenomatous polyposis), pregnancy and/or continuous treatment with NSAID, anti-coagulant or phosphodiesterase inhibitor. Furthermore, incomplete examination of the entire colon resulted in exclusion.

Patients were divided into 2 groups based on endoscopic findings and medical history: patients with present or history of CRN defined as either sessile serrate lesions (all types), high and low grade tubular adenomas, villous adenomas, tubule-villous adenomas and adenocarcinomas were termed CRN patients and patients without present nor history of CRN termed and served as controls, CTRL patients. A total of 73 patients were enrolled, hereof 53 CRN patients (Male/Female = 27/26) of which 5 were diagnosed with CRC (one patient had T3N1M0, while the others had T1N0M0) and remaining 20 were CTRL patients (Male/Female = 8/12). Mean age was 63 (50–78) in CRN patients and 61 (46–76) in CTRL patients. Twenty-eight patients in the CRN group and 5 patients in the CTRL group were regularly using medications e.g. anti-diabetics, anti-estrogens, anti-epileptics, anti-hypertensives, asthma inhalers, bisphosphonate, methotrexate, proton pump inhibitors, thyroid hormones, triptans, selective serotonin reuptake inhibitors, statins and xanthine oxidase inhibitors. An expected imbalance between patient groups was observed for comorbidities and medications. This diversity could have a potential impact on the obtained results.

### Ethics

The study protocol was approved by the Scientific Ethical Committee of Copenhagen (H-3-2013-107) and the Danish Data Protection Agency (BBH-2013-024, I-Suite no: 02342). The study was conducted in accordance with the Helsinki declaration. All participating patients gave written informed consent.

### Chemicals

SC 51322, PF 04418948, L-798,106, L-161,982, amiloride, theophylline, indomethacin, acetazolamide, bumetanide, ouabain as well as salts for Ringer’s solution were purchased from Sigma-Aldrich (Brøndby, Denmark). GW627368X, TCS 2510, and Sulprostone were purchased Santa Cruz Biotechnology (Texas, USA). ONO-DI004 and ONO-AE1-259 were kindly provided by Ono Pharmaceuticals Co., Ltd. (Osaka, Japan). All other chemicals were of analytical grade.

Selection of receptor agonists and antagonists was based on a thorough search of available literature, with a preference for compounds tested on human tissue.

### Biopsy extraction

All endoscopies and biopsy extractions took place at the Endoscopic Unit of Digestive Disease Center K, Bispebjerg Hospital, Nielsine Nielsens Vej 41K, 2400 Copenhagen NV, Denmark. Six endoscopic biopsies were obtained from each patient using standard biopsy forceps (Boston Scientific, Radial Jaw 4, large capacity). Biopsies were taken from macroscopically normal appearing sigmoid mucosa on retraction of the endoscope; about 30 cm orally from the anal verge and at least 10 cm from macroscopically abnormal appearing tissue.

Four biopsies allocated for functional studies, were immediately placed in an iced bicarbonate Ringer solution containing (in mM): Na^+^ (140), Cl^−^ (117), K^+^ (3.8), PO^−^_4_ (2.0), Mg^2+^ (0.5), Ca^2+^ (1.0), and HCO^−^_3_ [25], and transferred to the laboratory. The remaining biopsies were snap frozen in liquid nitrogen and stored at − 80 °C until further examination.

### Experimental methods

Two experimental methods were employed: functional studies in modified air suction Ussing (MUAS) chambers measuring short circuit current (SSC) and quantitative real-time polymerase chain reaction (qPCR).

#### Functional studies in MUAS-chambers

Four biopsies were mounted and oxygenated in MUAS-chambers after extraction as described by Larsen et al. [22] generally within 45 min after extraction. Biopsies were bathed on both sides with 10 mL Ringer, supplemented with 5.5 mM D-glucose. Temperature was maintained at 37.2 °C by water jackets. An automated voltage-clamp device continuously recorded SCC and slope conductance [22].

Experiments began after a stable basal SCC was obtained within 10 min after proper mounting. All experiments were initiated by addition of amiloride (20 µM, mucosal side) to inhibit electrogenic sodium absorption mediated through epithelial sodium channels and followed by theophylline (400 µM, serosal side) to inhibit phosphodiesterase-dependent cyclic adenosine monophosphate (cAMP) degradation. Finally, to eliminate endogenous prostaglandin synthesis, indomethacin (13 µM, serosal side) was added and incubated for 40 min.

Biopsies from 47 patients were treated with PGE_2_ and selective EP receptor agonists to investigate receptor function, Table 1. A single agonist was added in increasing concentrations (1 nM to 5 µM, serosal side) to each MUAS-chamber. The final agonist concentration step was followed by the addition of 5 µM PGE_2_, to elicit a maximal PGE_2_-induced response.

**Table 1:** Selected agonists and antagonists and applied antagonist concentrations for functional MUAS chamber experiments

Receptor subtype	Agonist	Antagonist with concentration	
EP1 receptor	ONO-DI004	SC 51322	2 µM
EP2 receptor	ONO-AE1-259	PF 04418948	3 µM
EP3 receptor	Sulprostone	L-798,106	500 nM
EP4 receptor	TCS 2510	L-161,982	2 µM
		GW627368X	5 µM

Biopsies from 26 patients were treated with selective EP receptor antagonists, Table 1. A combination of 3 antagonists was added to each MUAS chamber (serosal side), to single out and investigate the remaining non-inhibited EP receptor subtype. After antagonist incubation (45 min), cumulative doses of PGE_2_ were added (3 nM to 1 µM, serosal side). The EP4 receptor was also examined with another selective antagonist, GW627368X (GW-X, 5 µM, serosal side).

Experiments were terminated by the addition of acetazolamide, a carbonic anhydrase inhibitor (250 µM, serosal side), to measure HCO_3_^−^/H^+^-secretion, followed by bumetanide (25 µM, serosal side), to inhibit Na–K–Cl cotransporters and chloride secretion, and finally the Na^+^/K^+^-ATPase inhibitor ouabain (0.2 mM, serosal side) to assess and ensure tissue viability and data quality.

#### Quantitative real-time PCR

##### RNA isolation

Twenty biopsies, 10 from CRN and 10 from CTRL patients, were matched according to gender and used for further qPCR investigations. RNA was extracted from the biopsies using RNeasy Mini Kit (Qiagen, Copenhagen, Denmark). Following extraction, RNA samples were placed on ice and quantified using a Nanodrop Spectrophotometer (LabTech International) in accordance with the Minimum Information for Publication of Quantitative Real-Time PCR Experiments Guidelines (MIQE guidelines) [23].

##### qPCR analysis

RNA was reverse transcribed to cDNA using the nanoScript2 (Primerdesign Ltd., U.K.) according to the manufacturer’s protocol. Quantitative analysis of specific genes of interest within our cDNA samples was determined using Precision-iC SYBR green mastermix (Primerdesign Ltd.) with the CFX96 Real-Time PCR Detection System (Bio-Rad, Denmark). Duplicate reactions were performed in 20 μL volumes containing 10 μL Precision-iC SYBR green master mix, 300 nM primer (Primerdesign Ltd.), 15 ng cDNA and made up to 20 μL with nuclease-free water. The following cycling conditions were used: initial activation at 95 °C for 10 min, followed by 40 cycles of 95 °C for 15 s, and 60 °C for 1 min and data was collected during each cycling phase. Melt curve analysis, to ensure each primer set amplified a single, specific product, completed the protocol. Quantification cycle (Cq) values were determined using Bio-Rad CFX96 Manager 3.0 software and the single threshold mode.

The geNorm reference gene selection kit (Primerdesign Ltd.) was used to identify the most stable reference genes and to determine optimal number of reference genes required for reliable normalization of qPCR data in these tissue samples [24]. ß-actin and glyceraldehyde 3-phosphate dehydrogenase (GAPDH) were validated as the most stable reference genes in samples. The expression levels of genes of interest are expressed relative to the mean Cq value of the reference genes in each sample.

Primers were designed, synthesized and quality controlled by Primerdesign Ltd., Additional file 1: Table S1. The sequences for the reference genes ß-actin and GAPDH are commercially sensitive and therefore unavailable.

### Data analyses

The present study is exploratory and therefore not statistically powered for specific endpoints. If identical experiments were performed on several biopsies from the same patient, a mean value of parameter results was used. A comparison of parameter values between patient groups was performed by an unpaired t-test when standard deviations were equal, and a Welch’s t-test if unequal. Furthermore, normality was tested for data. Data are presented as mean ± SEM.

To assess agonists and receptors, data obtained from dose–response curves were analyzed with either a single-Michaelis–Menten model (srm) or a two-Michaelis–Menten receptor/site model (trm) using Sigmaplot 13.0 for Windows, Systat Software Inc. (USA/Canada). Outcome data were maximum SCC responses (R_Max_) and EC_50_ of these analyses.

All other statistics were performed using RStudio (Boston, USA), or GraphPad Prism (San Diego, USA) version 8 for the qPCR analysis. *P*-values < 0.05 were considered significant.

## Results

### High and low affinity EP receptors and nanomolar EP4 receptor potency

PGE_2_ stimulation increased SCC in both patient groups, even at concentrations as low as 1 nM, Additional file 1: Figs. S1 and S2. The EP4 agonist produced a similar sensitivity, demonstrating high potency in the low nanomolar range, Additional file 1: Fig. S1. Concentrations of 30 nM or higher were necessary to induce SCC increases when stimulating with the other selective EP-agonists, Additional file 1: Fig. S1. Moreover, 4 out of 22 biopsies exposed to the selective EP1 agonist showed no increase in SCC.

**Fig. 2:**
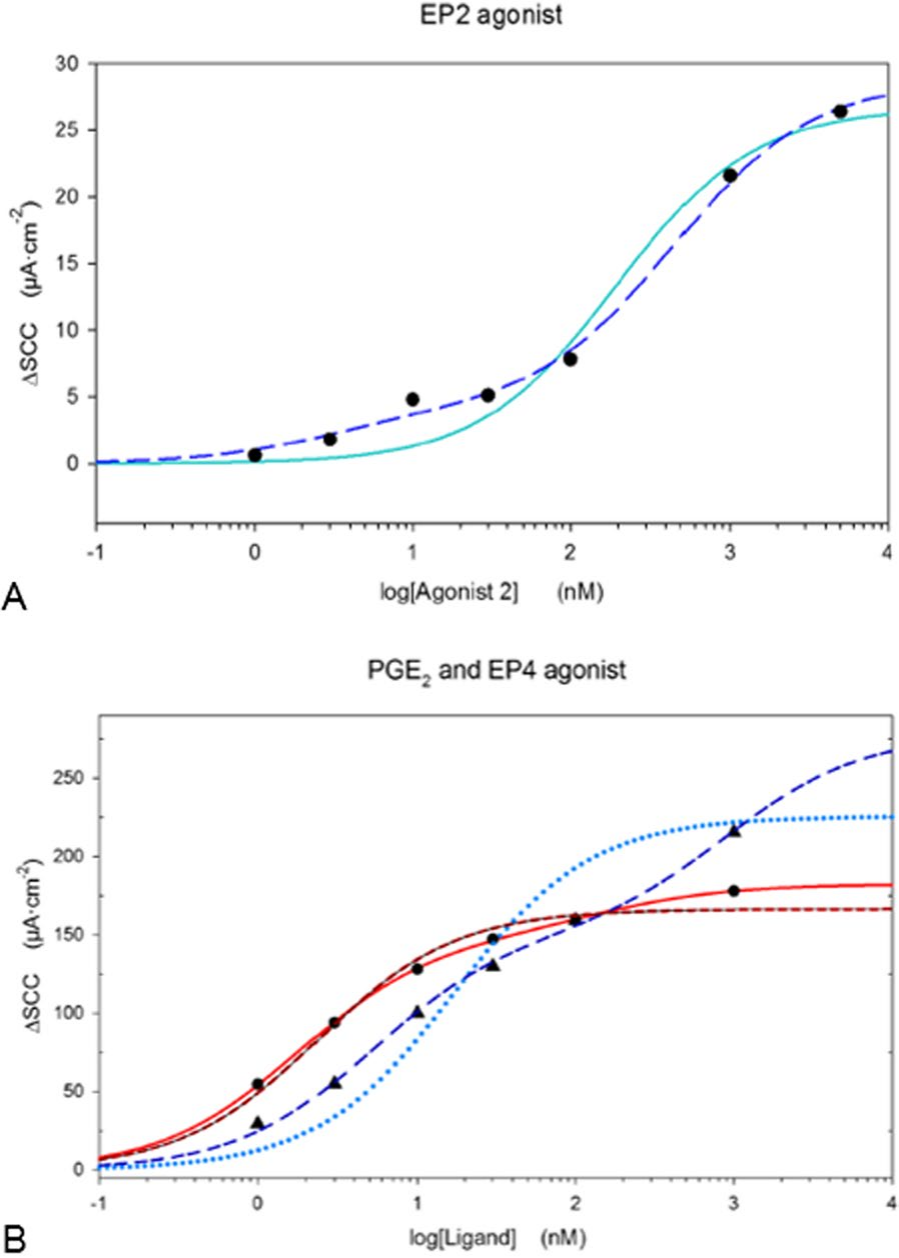
Dose–response curves of (**A**) EP2 agonist ONO-AE1-259 and (**B**) PGE_2_ and EP4 agonist, TCS 2510, experiments. X-axis: ligand concentrations scaled logarithmically. Y-axis: changes in SCC. **A**: Large dots (black) show increases in SCC as a response to increasing EP2 agonist concentrations. The unbroken line in cyan resembles single receptor model (srm) fitting, while the long dotted line in blue resembles two receptor model (trm) fitting. **B**: Triangles (black) indicate increases in SCC as a response to increasing PGE_2_ concentrations. Large dots (black) show increases in SCC as a response to increasing EP4 agonist concentrations. Dotted and long dotted lines (in blue colors) resemble single (srm) and two receptor model (trm) fitting for PGE_2_ respectively. The unbroken and the medium dotted lines (in red colors) show trm and srm respectively for EP4 agonist. The trm fits data points more closely

**Fig. 3:**
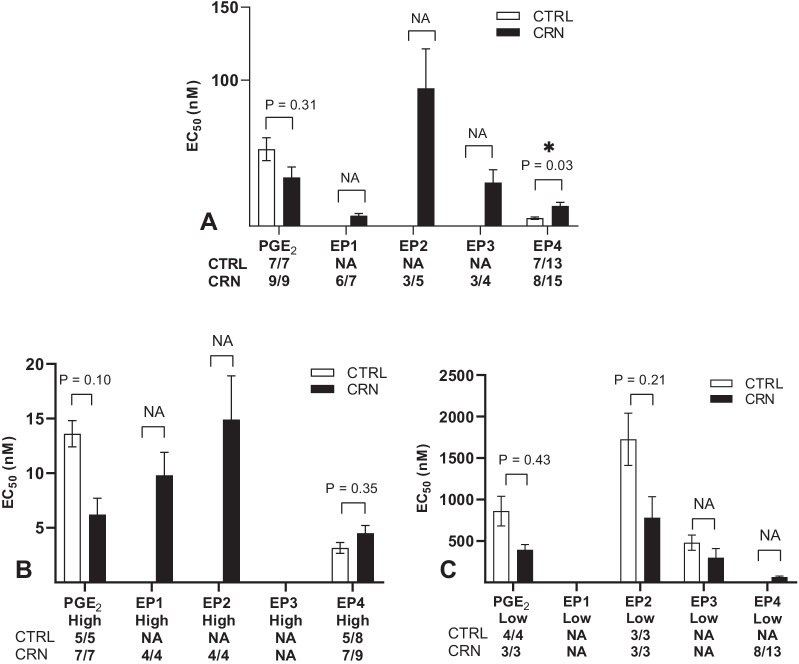
Calculated mean EC_50_ values of PGE_2_ and EP receptor agonists using (**A**) single receptor model (srm) equations and of (**B**) high and (**C**) low affinity receptors following PGE_2_ and EP receptor agonists stimulation using two receptor model equations (trm). Numbers under the graph show N/n, N = number of patients, n = number of biopsies, NA = not applicable due to insufficient N/n. Data are presented as means ± SEM. *p < 0.05

When applying Michaelis–Menten models (srm and trm) to data, a trm provided a better fit than the srm in most analyses of data from experiments with PGE_2_, and agonists for EP2 and EP4 receptor subtypes, Fig. 2. Accordingly, at least 2 types of EP receptors appear activated, a high and a low affinity receptor, with different EC_50_s separated by a factor up to 200 in single experiments, Fig. 3. Average separation factors of the receptors were 64 for PGE_2_ stimulation and 15 for the EP4 agonist, Fig. 3. In experiments using either the EP1 agonist or the EP3 agonist, trm equations did not fit convincingly. Mean EC_50_ values from both srm and trm analyses are summarized in Fig. 3. Using the srm, CRN patients demonstrated a higher EC_50_ related to stimulation with the EP4 agonist compared to CTRLs, Fig. 3A.

**Fig. 4:**
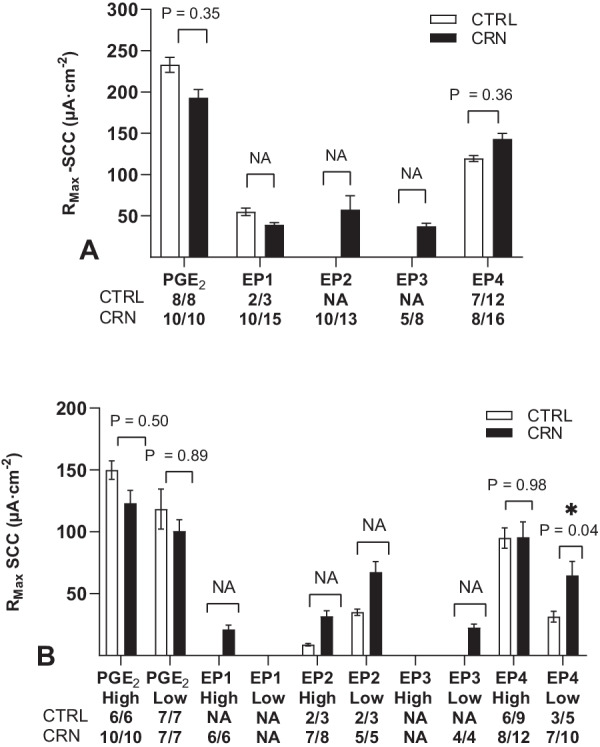
Calculated mean R_Max_ values displayed as µA·cm^−2^ from (**A**) single receptor models and (**B**) two receptor models upon biopsy stimulation with PGE_2_ or a selective EP receptor agonist. Numbers under the graph show N/n, N = number of patients, n = number of biopsies, NA = not applicable due to insufficient N/n. Data are presented as means ± SEM. *p < 0.05

Maximum SCC responses (R_Max_) computed from srm and trm are shown in Fig. 4. As PGE_2_ stimulates all EP receptors, R_Max_ was highest for PGE_2_ followed by the selective EP4 agonist eliciting approximately 50% and 75% of the PGE_2_ response in CTRL and CRN patients, respectively. The remaining EP-agonists had R_Max_ means ranging between 20 and 30% of the PGE_2_ response. Finally, R_Max_ was significantly increased for low affinity receptors in EP4 agonist studies (trm) in CRN patients, Fig. 4B.

### Selective EP antagonists are unsuitable for determining EP receptor subtypes

Forty-one biopsies from 26 patients were exposed to EP antagonist cocktails, intended to inhibit all but one of the 4 EP receptor subtypes, followed by increasing PGE_2_ concentrations. To our surprise, we recorded sizable SCC increases upon ensuing PGE_2_ stimulation, even in the low nanomolar range, regardless of antagonist combination as well as in the presence of all 4 EP receptor antagonists, data not shown. These data indicate a lack of irreversible and/or competitive inhibition by all the 4 selective EP antagonists. Thus, with the present study design and protocol, none of the employed selective antagonists acted as expected.

### Competitive antagonism between EP4 receptor antagonist GW-X and PGE_2_

**Fig. 5:**
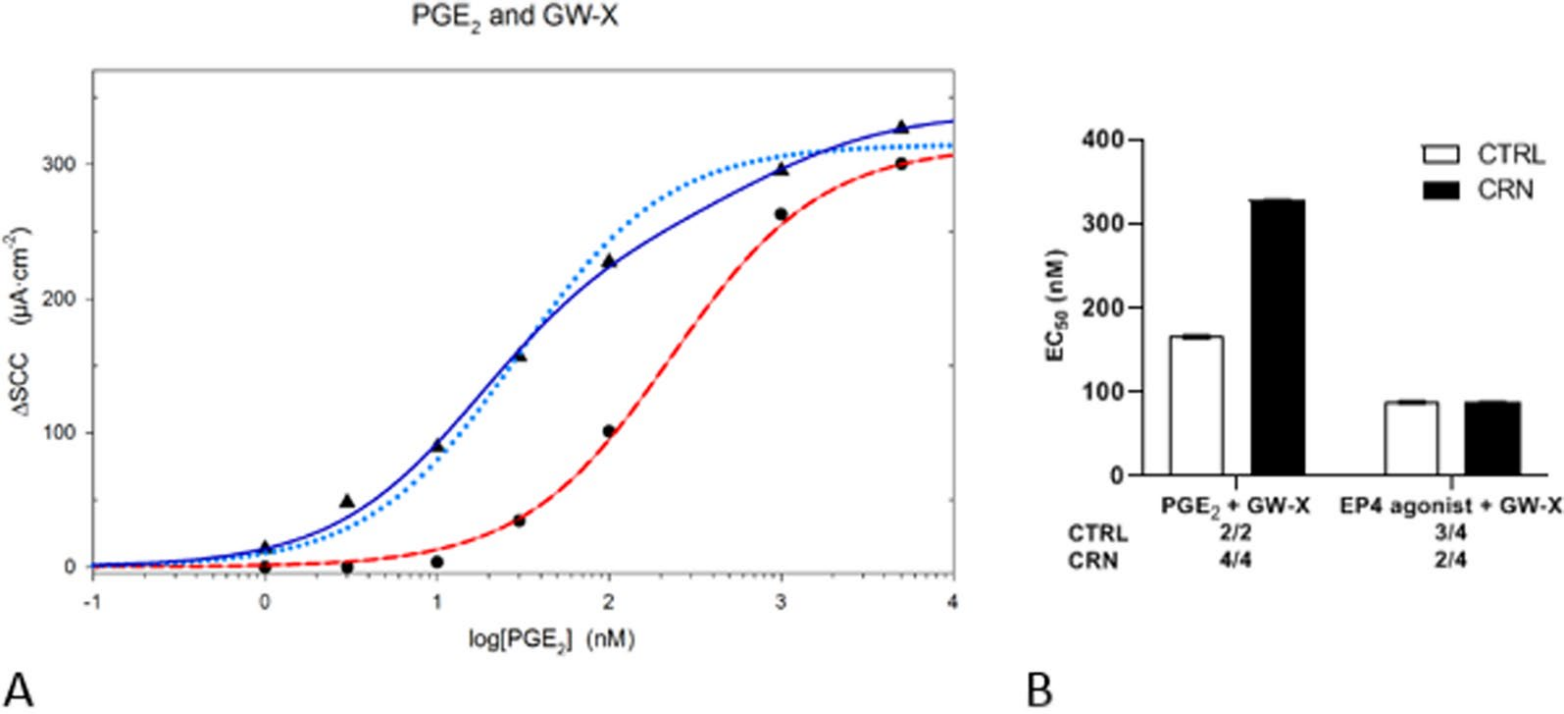
Dose–response curves of PGE_2_ stimulation with and without EP4 antagonist GW627368X (GW-X) and calculated mean EC_50_ values. **A**: X-axis: PGE_2_ concentrations scaled logarithmically. Y-axis: changes in SCC. Triangles (black) show increases in SCC as a response to PGE_2_ doses without the addition of GW-X. Big dots (black) show increases in SCC in the presence of EP4 antagonist GW-X followed by PGE_2_ stimulation. The small dotted and the unbroken line (blue colors) resemble single (srm) and two receptor model (trm) fitting. Long dotted line (red) shows srm for experiments with GW-X, trm could not be calculated. **B**: Mean EC_50_ (nM) values of PGE_2_ and EP4 agonist TCS 2510 following inhibition with GW627368X (GW-X), calculated from single receptor model (srm) equations**.** Numbers under the graph show N/n, N = number of patients, n = number of biopsies. Data are presented as means ± SEM

Additional experiments were performed with only the selective EP4 antagonist GW-X, added prior to stimulation with PGE_2_. Figure 5 A shows the rightward shift induced by GW-X on PGE_2_ dose–response curves. The effect of GW-X demonstrates a competitive inhibition of PGE_2_ in the low nanomolar concentration range. Moreover, high PGE_2_ concentrations elicited about the same maximal increase in SCC regardless of GW-X addition, further supporting simple competitive antagonism between GW-X and PGE_2_. An agonist-based Cheng-Prusoff analysis of the PGE_2_-GW-X interactions resulted in an IC_50_ of 210 nM for GW-X, see Additional file 1: Data S1 and Fig. 5 B. To run a t-test for reliable judgement of differences in mean EC_50_s for GW-X between patient groups, more experiments are required.

### EP1 and EP2 receptor subtypes are upregulated in CRN patients

**Fig. 6:**
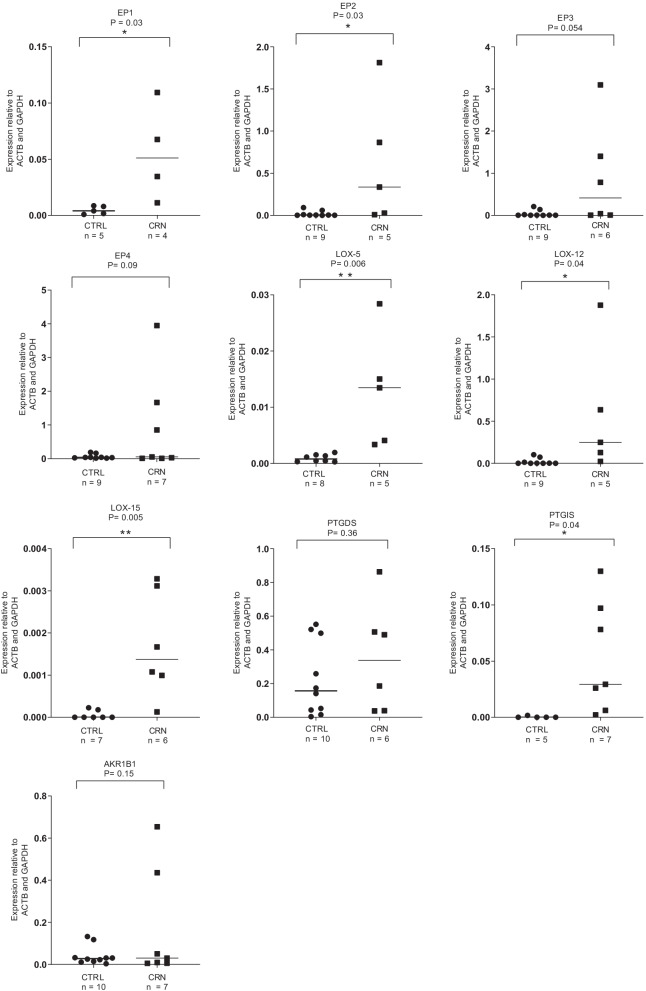
Expression levels of investigated enzymes and receptors. Expression of EP1, EP2 5-LOX, 12-LOX, 15-LOX as well as PTGIS are significantly higher in CRN patients. Expression levels are relative to ß-actin and GAPDH. Data are presented as means ± SEM. *p < 0.05 and **p < 0.01

mRNA expression levels of EP1 and EP2 were elevated in CRN patients compared to CTRLs, Fig. 6. EP3 and EP4 mRNA expression showed a trend of elevation in CRN patients.

### Enzymes related to the COX and LOX pathways are upregulated in CRN patients

All investigated LOX enzymes (5-LOX, 12-LOX, and 15- LOX) demonstrated elevated levels of mRNA in CRN patients compared to CTRLs, Fig. 6. Moreover, the expression of PTGIS was significantly upregulated in the CRN group, whereas expression levels of PTGDS and ARK1B1 were unaltered, Fig. 6.

## Discussion

In the present study, we identified several differences in normal-appearing colonic mucosa from CRN patients, supporting the hypothesis of aberrations in enzymes and receptors of the eicosanoid pathway.

Independently of CRN history, we demonstrate that EP receptors bind PGE_2_ with 2 different affinities indicating the presence of high and low affinity EP receptor subtypes. Furthermore, we observed similar mucosal responses to selective EP2 and EP4 receptor agonists. Assuming selectivity of these compounds towards their receptors, our data suggest presence of both a high affinity EP4 and a low affinity EP2 receptor subtype [25, 26]. High and low affinity EP receptors in human colonic mucosa have been reported previously, but not investigated further [27, 28].

Our experiments identified the EP4 receptor to be the EP receptor subtype with the highest secretory response in the colon, which is consistent with existing reports [28, 29]. Furthermore, based on experiments with the highly selective EP4 receptor agonist TC 2510 [26], our data suggest a presence of both high and low affinity EP4 receptors with associated higher mean potencies and lower mean efficiencies compared to PGE_2_. Meanwhile, the existence of 2 EP4 receptors was not corroborated by experiments with the selective EP4 receptor antagonist, GW-X, which was effective in human colonic mucosa previously [28]. GW-X eliminated the biphasic PGE_2_ dose–response curve, resulting in a single receptor dose–response curve. This may be explained as a surmountable rightward potency-shift for a single EP4 high affinity receptor, moving it closer to the potency of the low affinity receptor(s) in the presence of GW-X, maintaining a combined efficiency at high concentrations of PGE_2_ with no antagonist present.

Stimulation of the EP4 subtype receptor is well documented as an important immunosuppressive trigger in the CRC microenvironment [30]. Accordingly, several interventional clinical phase-1 studies with focus on CRC have been initiated with newly developed EP4 antagonists [31], and recently another trial, testing an EP4 antagonist in metastatic CRC patients, has proceeded to phase II (NCT05205330). Furthermore, another study points to a carcinogenic mechanism involving pericryptal COX-2-expressing fibroblasts, which exert paracrine control over tumor-initiating stem cells via a COX-2 and PGE2–EP4–Yap signaling pathway [32, 33].

Taken together and respecting the relative few subjects in the present study, our findings support presence of high sensitivity for PGE_2_ in even normal appearing colonic mucosa.

Separate additions of single selective EP antagonists did not change the ensuing PGE_2_-induced SCC. Whether the PGE_2_-induced SCC increases reflect remaining secretion of incompletely inhibited EP receptor subtype(s) or resemble PGE_2_-induced secretion by other prostanoid receptors cannot be ascertained. Surprisingly, employed EP receptor antagonists, except for GW-X, were not useful in the present study. Our findings have not been tested under the same in vivo conditions by others, so the results await confirmation from other laboratories.

Our mRNA expression studies revealed increased expressions of receptor subtypes EP1 and EP2 in CRN patients. We, as others, have investigated EP receptor expression levels in human colonic tissue previously [34, 35]. The mRNA expression levels for EP1, EP2 and EP3 in this study are at variance with a former study from our laboratory [34]. Since identical primers against the subtype receptors were used in the 2 studies, presently the only recognized difference in study design were the number of reference genes, as two reference genes where used in the present study, while only one was used in the study by Petersen et al. Beside this our only other explanation for the deviation in results, is a greater variance in the general population of humans undetectable in small scale studies. Thus, our results should be taken as preliminary indication and be confirmed in much larger cohorts.

We found PTGIS expression to be upregulated in CRN patients. Previous expression studies of PTGIS/PGI_2_ in CRC patients have been ambiguous. One study found decreased PGI_2_ levels using radioimmunoassay in CRC patients [36]. Conversely, Lichao et al. found weak or no staining of PTGIS in normal tissue (corresponding to our biopsies from CRN patients) in microarray expression studies, while PTGIS expression was detected in CRC patients and increased in CRC patients with liver metastasis [37]. Merging results, we hypothesize a stepwise increase relationship in PTGIS expression and the degree of colonic mucosa dysplasia and risk of liver metastasis.

All tested LOX enzymes had higher mRNA expression levels in colonic mucosa from CRN patients. For 5-LOX and 12-LOX, this is consistent with the bulk of literature. Both enzymes elicit key pro-inflammatory and pro-tumorigenic downstream functions and are upregulated in human colon adenomas and adenocarcinomas [16, 38, 39]. Our results suggest that an upregulation of the LOX pathway is already present in normal appearing colonic mucosa from CRN patients. As such, 5-LOX and/or 12-LOX, enzyme expression might possess the potential of becoming an early predictive biomarker of CRN development.

Both 15-LOX isoforms are considered anti-tumorigenic and especially 15-LOX-1 and its product 13(S)-HODE appear tumor protective and downregulated in CRC tissue [17, 39]. Our employed 15-LOX primer unfortunately did not differentiate between the 2 isoforms. In contrast to previous studies, we observed increased 15-LOX expression in the mucosa of CRN patients. Given that we only investigated normal-appearing mucosa, the observed upregulated expression of 15-LOX might be a compensatory effect before mucosal cells become neoplastic. It would be interesting to further track the expression of 15-LOX, to determine whether the expression is suppressed as the cells become carcinogenic.

Several studies have addressed, documented, and discussed aberrant gene expression in tumor-adjacent colonic mucosa in relation to so-called field cancerization (tumor or environmental signaling) and mutator pathways based on tumor-induced mutations in DNA-repair genes, epigenetic methylation, genetic instability and tumor suppressor entities [18, 20, 40–45]. Furthermore, some aspects of such hypotheses are separated out as etiological factors termed ‘etiological field effects’ involving lifestyle, food mutagens, the gut microbiome, as well as environmental, hormonal, and genetic factors [43]. With an aspect of possible predisposition markers as in this study, only few studies have compared gene expression levels between normal colonic mucosa from control patients and macroscopically normal tumor-adjacent mucosa (> 10 cm tumor-distance), from CRN patients [34, 46, 47].

Lastly, it should be stressed, that our study is observational with a limited number of participants. Thus, our findings of aberrant enzyme and receptor expressions must be taken as indicators of possible predisposing factors, while confirmation of our observed statistically significant deviations requires much larger cohorts. In future studies, mRNA results should also be verified with other methods such as for example immunoblotting. Furthermore it would be preferable to get more cell type/molecular information per biopsy, as this is, even though well-known and accepted, a limitation to the study design.

## Conclusions

Normal appearing colonic mucosa from patients with history of CRN demonstrates altered enzymatic expression of the eicosanoid pathway. Our data suggests a likely gene-based predisposition for early disease development. Furthermore, PGE_2_ did activate EP receptors with different affinity including a high affinity EP4 receptor with nanomolar potency to PGE_2_. Whether this highly sensitive EP4 receptor is tumorigenic and as such could be targeted in CRN management remains to be clarified. The observed aberrant gene expressions,

## Supplementary Information

**Additional file 1.**Supplementary study data.

## Data Availability

The datasets used and/or analysed during the current study are available from the corresponding author on reasonable request.

## Abbreviations

AA: Arachidonic acid
AKR1B1: Aldoketoreductase 1B1
ASA: Acetylsalicylic acid
COX-1: Cyclooxygenase 1
COX-2: Cyclooxygenase 2
Cq: Quantification cycle
CRC: Colorectal cancer
CRN: Colorectal neoplasia
cAMP: Cyclic adenosine monophosphate
EP1: Prostaglandin E2 receptor subtype 1
EP2: Prostaglandin E2 receptor subtype 2
EP3: Prostaglandin E2 receptor subtype 3
EP4: Prostaglandin E2 receptor subtype 4
GAPDH: Glyceraldehyde 3-phosphate dehydrogenase
GW-X: GW627368X
HETE: Hydroxyeicosatetraenoic acids
HPETE: Hydroperoxyl-eicosatetraenoic acids
MIQE Guidelines: Minimum Information for Publication of Quantitative Real-Time PCR Experiments Guidelines
MUAS-chamber: Modified Ussing air suction chamber
NSAID: Non-steroid anti-inflammatory drug
PTGDS: Prostaglandin D2 synthase
PGE_2_: Prostaglandin E2
PTGIS: Prostaglandin-I2 synthase
qPCR: Quantitative real-time polymerase chain reaction
SCC: Short circuit current
