# Correction: Nanomolar EP4 receptor potency and expression of eicosanoid-related enzymes in normal appearing colonic mucosa from patients with colorectal neoplasia

**DOI:** 10.1186/s12876-022-02339-1

**Published:** 2022-06-17

**Authors:** Ulrike Ries Feddersen, Sebastian Kjærgaard Hendel, Mark Alexander Berner-Hansen, Thomas Andrew Jepps, Mark Berner-Hansen, Niels Bindslev

**Affiliations:** 1 Digestive Disease Center, Bispebjerg Hospital, 2400 Copenhagen NV, Denmark; 2 Department of Biomedical Sciences, University of Copenhagen, 2200 Copenhagen N, Denmark

## Correction to: BMC Gastroenterology (2022) 22:234 10.1186/s12876-022-02311-z

After publication of this article [1], the authors reported that the last sentence of the Conclusions section “The observed aberrant gene expressions,” should have read: “The observed aberrant gene expressions, as an individual predisposition for early tumorigenesis, is still an open question.”

The original article [1] has been updated.
