# Supplementary material for: Nanomolar EP4 receptor potency and expression of eicosanoid-related enzymes in normal appearing colonic mucosa from patients with colorectal neoplasia
Source: BMC Gastroenterol. 2022 May 12;22:234. doi: 10.1186/s12876-022-02311-z (PMC9097415; doi:10.1186/s12876-022-02311-z)
Supplement: Supplementary file 1 — Additional file 1.Supplementary study data. [file 12876_2022_2311_MOESM1_ESM.docx]

Additional file 1

**Table S1.** Employed qPCR primers against genes of interest.

| Gene | Forward | Reverse |
| --- | --- | --- |
| EP1 | 5’-ATCTGCTGGAGCCCAATGC-3’ | 5’-GATCTGGTTCCAGGAGGCAA-3’ |
| EP2 | 5’-CAGACCCTGGTGGCACTG-3’ | 5’-CGAAGAGCATGAGCATCGTG-3’ |
| EP3 | 5’-TCAATCAGACATCAGTTGAGCAC-3’ | 5’-TTTCTTAACAGCAGGTAAACCCAA-3’ |
| EP4 | 5’-AGTTTGGAGCGAGAAGTCAGTA-3’ | 5’-GCGGCAGAAGAGGCATTTG-3’ |
| PTGDS | 5’-GGGCTTCACAGAGGATACCA-3’ | 5’-CCCTGGGGAGTCCTATTGTT-3’ |
| PTGIS | 5’-AGACGGGCGAGAATTCAACC-3’ | 5’-CGTCAGGGTTCAGGAATTCGG-3’ |
| AKR1B1 | 5’-GGATGAGTCGGGCAATGTGG-3’ | 5’-TGGAGATGGTTGAAGTTGGAGATG-3’ |
| LOX-5 | 5-ACCTGTTCATCAACCGCTTCA-3’ | 5’-GGTCTTCCTGCCAGTGATTCA-3’ |
| LOX-12 | 5’- CTCCTGGAACTGCCTAGAAGAC-3’ | 5’-CTGGTAGCTGAACAACTCATCATC-3’ |
| LOX-15 | 5’-ATCTTCTGAGGGGACACTTGA-3’ | 5’-GTATCGCAGGTGGGGAATTATA-3’ |

**Figure S1.**


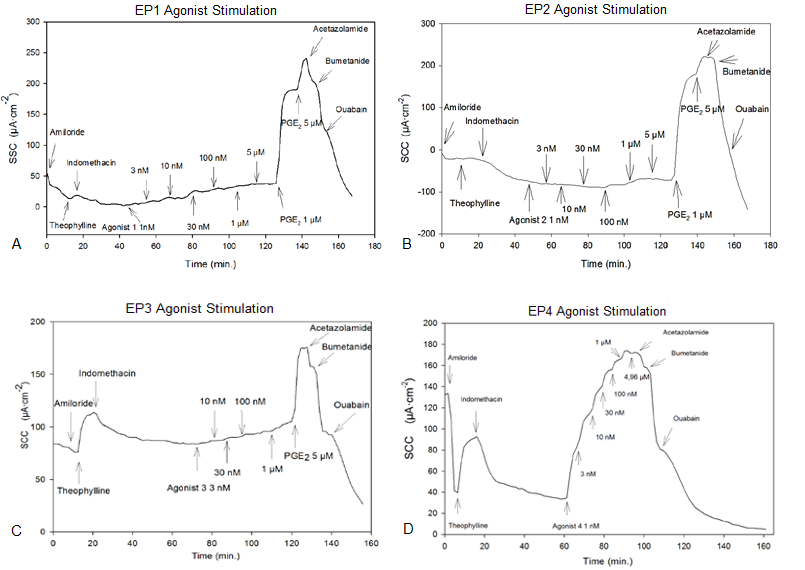


**Figure S1.** Examples of functional MUAS-chamber experiments.

Short-circuit current (SCC, µA·cm^⁻2^) measurements from normal appearing human colonic mucosa biopsies in 4 patients with CRN, showing concentration response analysis of EP 1 agonist, ONO-DI004, stimulation (A), a EP2 agonist, ONO-AE1-259, (B) a EP3 agonist, Sulprostone, stimulation (C) and a EP4 agonist, TCS 2510 stimulation (D). Biopsies were exposed initially to amiloride (sodium absorption inhibitor, 20 µM), then theophylline (non-specific phosphodiesterase inhibitor, 400 µM), then indomethacin (non-specific COX inhibitor, 13 µM) followed by one of the agonists in increasing concentrations (1 nM – 5 µM). If only small SCC increases were measured, single doses of PGE_2_ were added. Ultimately acetazolamide (carbonic anhydrase inhibitor, 250 µM), bumetanide (Na-K-Cl-cotransporter inhibitor, 25 µM) and ouabain (Na^+^/K^+^-ATPase inhibitor, 200 µM).


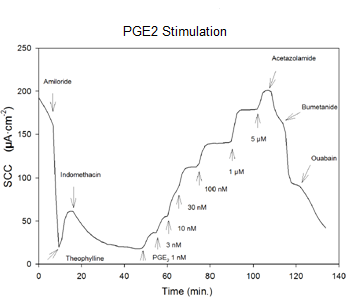
**Figure S2.**

**Figure S2.** Examples of a functional MUAS-chamber experiment with PGE_2_. Short-circuit current (SCC, µA·cm^⁻2^) measurements from normal appearing human colonic mucosa biopsies in one patients with CRN, showing concentration response analysis of PGE_2_. Biopsies were exposed initially to amiloride (sodium absorption inhibitor, 20 µM), then theophylline (non-specific phosphodiesterase inhibitor, 400 µM), then indomethacin (non-specific COX inhibitor, 13 µM) followed by PGE_2_ in increasing concentrations (1 nM – 5 µM). Ultimately acetazolamide (carbonic anhydrase inhibitor, 250 µM), bumetanide (Na-K-Cl-cotransporter inhibitor, 25 µM) and ouabain (Na^+^/K^+^-ATPase inhibitor, 200 µM).

**Data S1.**

Calculation of IC_50_ for EP4 antagonist GW-X

From 2 agonist dose­-response curves, one determined in the absence of a competitive antagonist, the other in its presence at only a single concentration, it is possible to calculate the antagonist apparent half inhibition dissociation constant at equilibrium, IC_50_. The parameters necessary for this, are the agonist EC_50_s in the absence and presence of the antagonist and a knowledge of the used antagonist concentration [I].

In simple Michaelis-Menten kinetics, without an antagonist, the apparent dissociation constant for an agonist is:

$\text{EC}_{50}^{0} \text{equal} \text{to} \text{K}_{\text{ss}}^{0}$, the 50 % activation constant in which the first subscript *s* designates a primary site for an agonist, and the agonist is designated with the second subscript *s.*

In simple competitive antagonism the apparent dissociation constant for the agonist is: $\text{EC}_{50}^{\text{anta}} \text{equal} \text{to} \text{K}_{\text{ss}}^{0}\cdot\left(\text{1+}\frac{\left[\text{I}\right]}{\text{K}_{\text{si}}}\right)$

IC_50_ is equal to K_si_, in which the first subscript *s* designates the primary site and the second subscript *i* designates an antagonist bound at the primary binding site. IC_50_ can be extracted from ratios of obtained agonist equilibrium constants:

$$\frac{\text{EC}_{50}^{\text{anta}}}{\text{EC}_{50}^{0}} \text{=} \text{K}_{\text{ss}}^{0}\cdot\frac{\text{1+}\frac{\left[\text{I}\right]}{\text{K}_{_{\text{si}}}}}{\text{K}_{\text{ss}}^{0}} \Leftrightarrow\text{K}_{\text{si}} \text{=} \text{IC}_{50} \text{=} \frac{\left[\text{I}\right]}{\left[\left(\frac{\text{EC}_{50}^{\text{anta}}}{\text{EC}_{50}^{0}}\right)\text{-1}\right]}$$

EC_50_ ratios,$\frac{\text{EC}_{50}^{\text{anta}}}{\text{EC}_{50}^{0}}$, can be calculated with values taken from figure 5 and table 3:

**Table S3. EC_50_ ratios based on 4 protocols**

Of these EC_50_ ratios, a mean value can be set at approximately 25.

With this and [I] = [GW-X] = 5 µM, we can calculate IC_50_:

$$\begin{matrix} \text{IC}_{50}\left(\text{= K}_{\text{si}}\right) \text{for} \text{GW-X} \text{:} 25 \text{=} \text{1+}\frac{\left[\text{I}\right]}{\text{K}_{\text{si}}} \\ \text{IC}_{50} \text{for} \text{GW-X} \text{=} 210 \text{nM} (n = 14) \end{matrix}$$

Thus, IC_50_ for antagonist GW-X is around 210 nM, based on a total of 14 individual experiments. The values for GW-X EC_50_ in table supplement 2 emerge nearly independent of the used agonists (PGE_2_ and TCS 2510) and patient groups (CRN and CTRL). This indicates that GW-X binds to a single receptor-site different from the agonist site *s*, equal to type II competitive antagonism with an apparent half inhibition dissociation constant at equilibrium, equal to K_ii_ (Reference 48 manuscript, DOI:10.4324/9781315159782).
